# Total laparoscopic excision of retroperitoneal ganglioneuroma: A case report

**DOI:** 10.1016/j.ijscr.2021.106053

**Published:** 2021-05-29

**Authors:** Mai Kitazawa, Nobuhisa Matsuhashi, Takeharu Imai, Yoshinori Iwata, Takao Takahashi, Kazuhiro Yoshida

**Affiliations:** Department of Surgical Oncology, Gifu University School of Medicine, Gifu University, 1-1 Yanagido, Gifu 501-1194, Japan.

**Keywords:** Ganglioneuroma, Retroperitoneal tumor, Laparoscopic surgery

## Abstract

**Introduction:**

Ganglioneuromas are rare benign tumors originating from neural crests and typically affect young adults. The most frequent locations are the posterior mediastinum, retroperitoneum and adrenal gland. In general, retroperitoneal ganglioneuromas are discovered incidentally or by mass effect. In the literature, the number of retroperitoneal masses reported is quite limited. We report a case of laparoscopic excision of a retroperitoneal ganglioneuroma.

**Presentation of case:**

The patient was a 40-year-old woman who visited a nearby clinic with anorexia and vomiting. She was referred to our hospital after the detection of an abdominal mass.

Enhanced computed tomography(CT) showed a lobule mass of 107 × 42 mm in size, with internal inhomogeneity and mild delayed enhancement on the retroperitoneal side of the left abdominal lesion. Magnetic resonance imaging(MRI) showed a mass with low intensity and partial high intensity on T2 weighted Image (T2WI). In addition, positron emission tomography CT(PET-CT) detected slight fluorodeoxyglucose (FDG) accumulation (standardized uptake value(SUV) max: 3.01) in the same lesion. Based on these findings, we suspected a retroperitoneal tumor. Laparoscopic excision was performed via 5 ports. The extracted tissue was a well-defined mass of 110 × 70 mm. The tumor in our case exceeded 10 cm.

The pathological diagnosis was ganglioneuroma, with no obvious malignancy.

**Discussion:**

It was suggested that adaptation of laparoscopic surgery should be considered based on the observation of organ invasion or vessel invasion and adhesion around the tumor, rather than based on the diameter of the tumor.

**Conclusion:**

This approach is less invasive than conventional laparotomy methods and achieves good cosmetic outcomes. Thus, totally laparoscopic procedures should be considered more often for the treatment of retroperitoneal tumors.

## Highlights

• Retroperitoneal tumor of >10 cm in size is usually treated by laparotomy surgery.
• However, totally laparoscopic approach can be performed with good outcomes.
• Adaptation of laparoscopic surgery should be considered based on organ or vessel invasion and adhesion around the tumor.
• This approach is less invasive and achieves good cosmetic outcomes.
• Totally laparoscopic surgery should be considered for retroperitoneal tumor excisions.

## 1 Introduction

Ganglioneuromas are rare benign tumors originating from neural crests and typically affect young adults. The most frequent locations are the posterior mediastinum, retroperitoneum and adrenal gland. In general, retroperitoneal ganglioneuromas are discovered incidentally or by mass effect. US and CT imaging may suggest the diagnosis, while MRI findings can be specific for ganglioneuroma. In the literature, the number of retroperitoneal masses reported is quite limited. We report a case of laparoscopic excision of a retroperitoneal ganglioneuroma.

This work has been reported in line with the SCARE criteria [1)].

## 2 Case presentation

**Fig. 1:**
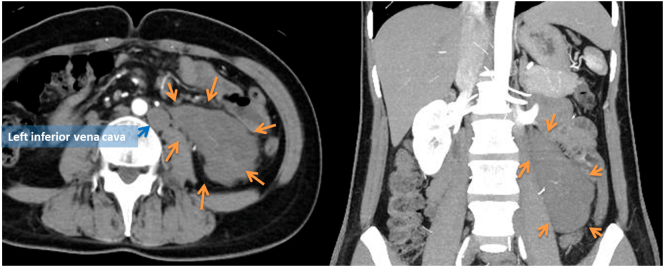
Using contrast-enhanced CT, the tumor was located in the left pararenal cavity to the retroperitoneum. The shape of the tumor was elongated and lobulated with a major axis of 10 cm. This patient had a duplicated inferior vena cava, of which there was no adipose tissue between the left inferior vena cava and the tumor.

**Fig. 2:**
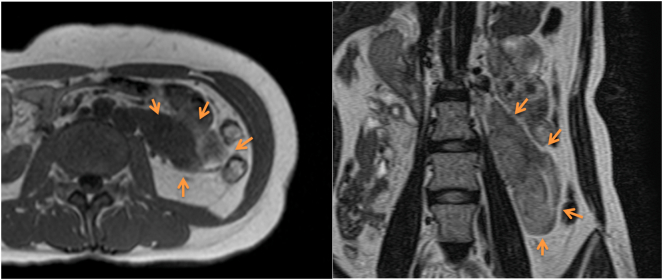
The tumor showed low signal intensity on T2 weighted image, which was located in retroperitoneal lesion.

**Fig. 3:**
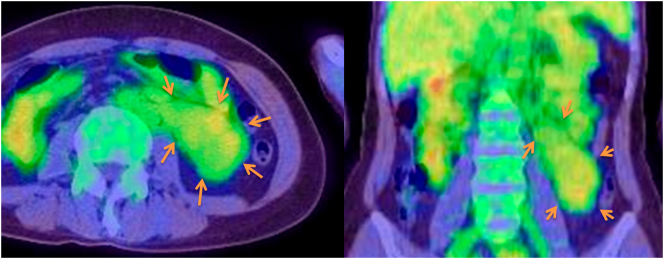
On PET-CT, the tumor showed slight FDG accumulation (SUVmax 3.01) in the left pararenal space. The preoperative diagnosis was retroperitoneal tumor (suspected desmoid tumor).

A 40-year-old previously healthy woman complaining of slight nausea, vomiting, and anorexia was referred to our hospital. Physical findings revealed a flat abdomen that was soft, not tender, and no palpable mass. CT and MRI examination showed a left abdominal lesion on the retroperitoneal side (Fig. 1, Fig. 2). CT showed a lobule mass of 107 × 42 mm in size, with internal inhomogeneity and mild delayed enhancement on the retroperitoneal side of the left abdominal lesion. MRI showed a mass with low intensity and partial high intensity on T2WI·In addition, PET-CT detected slight FDG accumulation (SUV max: 3.01) in the same lesion (Fig. 3).

A retroperitoneal tumor, particularly a desmoid tumor, was suspected based on the abdominal MRI, abdominal CT, and PET-CT findings. A solitary fibrous tumor, malignant lymphoma, fatty tumor and neurogenic tumor were considered as differential diagnoses. Thus, total laparoscopic surgery was performed to resect the retroperitoneal tumor.

**Fig. 4:**
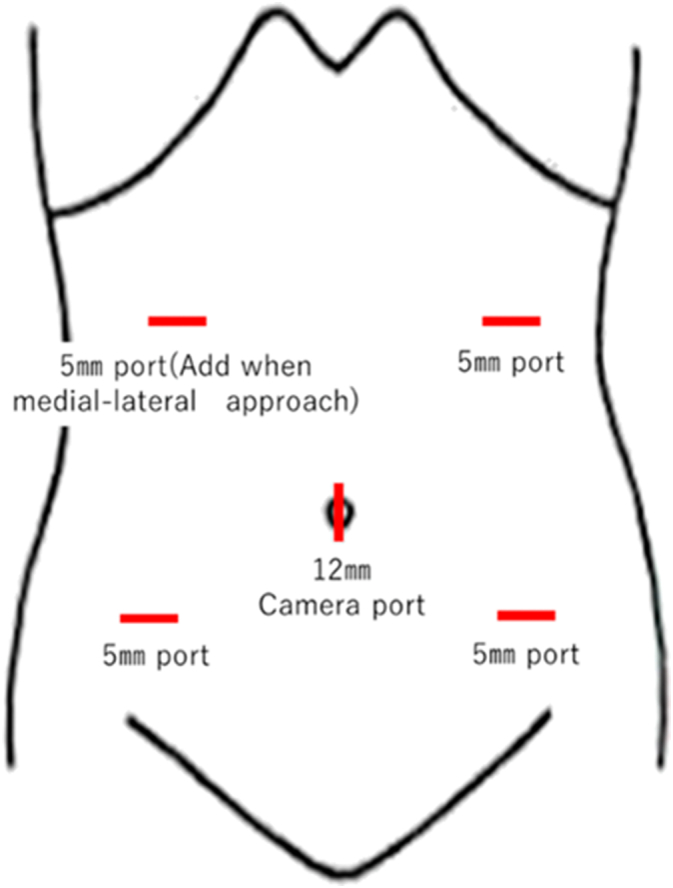
We started with four ports at first, and we added one more port when we performed medial-lateral approach.

**Fig. 5:**
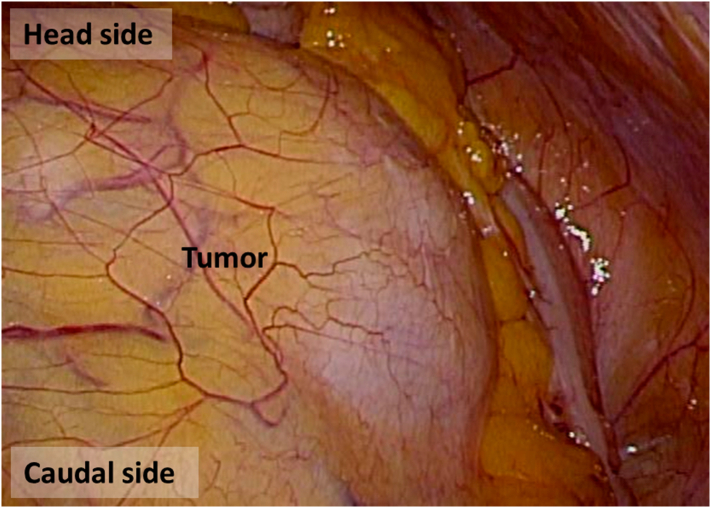
The tumor was located from the lower pole of the left kidney to the renal pelvis.

**Fig. 6:**
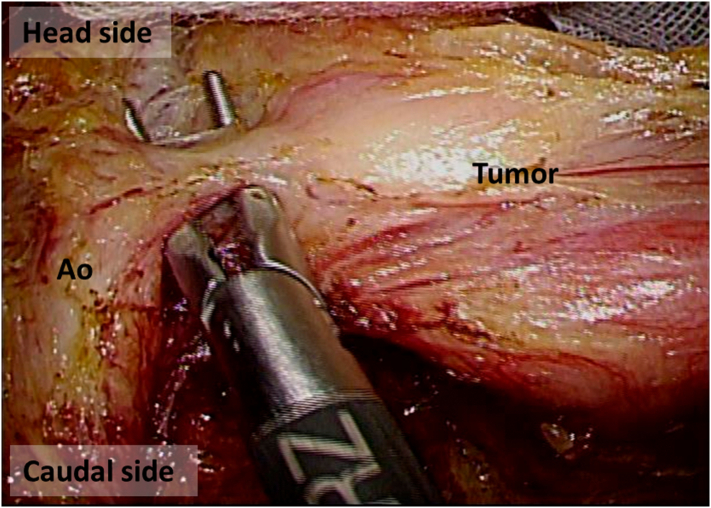
The tumor was adjacent to the left side of the aorta and the left lower vena cava, but no invasion was observed.

**Fig. 7:**
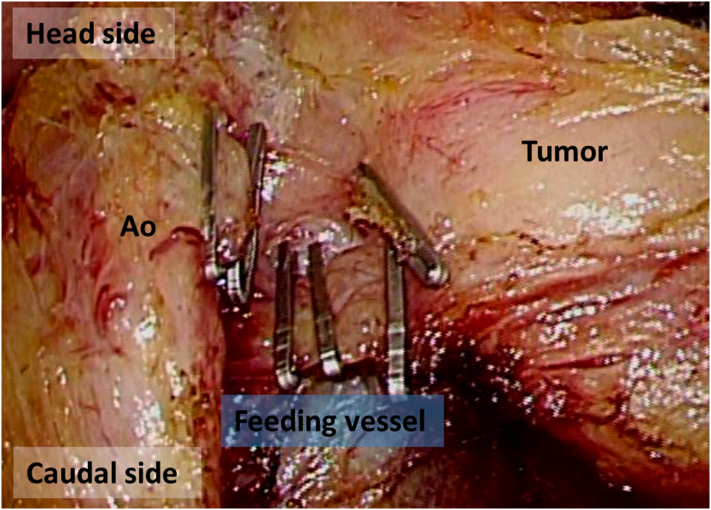
Two feeding arteries were flowing into the tumor from the aorta and the IMA. Each artery was clipped. In addition, two feeding veins flowing into the vena cava were clipped.

The operation was performed as follows. First, laparoscopic exploration was performed with five ports after inducing pneumoperitoneum (Fig. 4). Secondly, mobilization of the jejunum and the ileum was conducted in the right-head-ventral side position (Fig. 5). This mobilization was indicated to provide an optimal surgical view of the tumor. Dissection of the left side of the colon was performed using a medial-lateral retroperitoneal approach Thirdly, dissection of the tumor around the inferior mesenteric artery was performed. The tumor was adjacent to the left side of the aorta and the left lower vena cava, but no invasion was observed, and two feeding arteries were flowing into the tumor from the aorta and the IMA. Each artery was clipped (Fig. 6, Fig. 7). In addition, two feeding veins flowing into the vena cava were clipped. The tumor specimen was removed via the umbilical wound.

The operative time was 124 min, and the operating blood loss was approximately 5 mL.

**Fig. 8:**
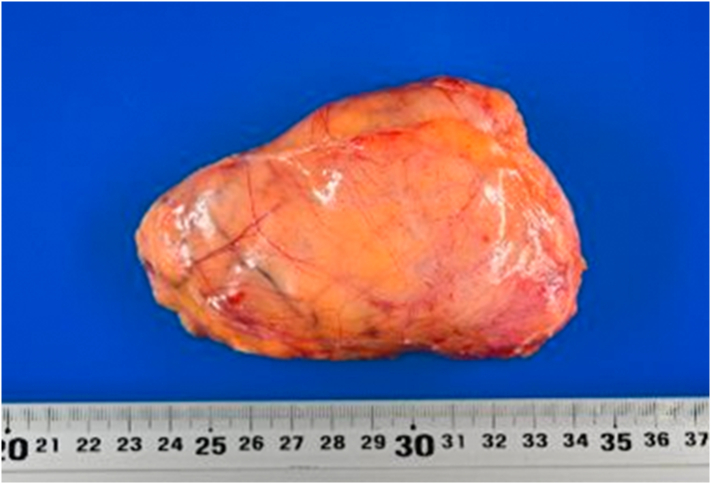
The tumor had a well-defined border with the surroundings and was covered with a fiber coat.

**Fig. 9:**
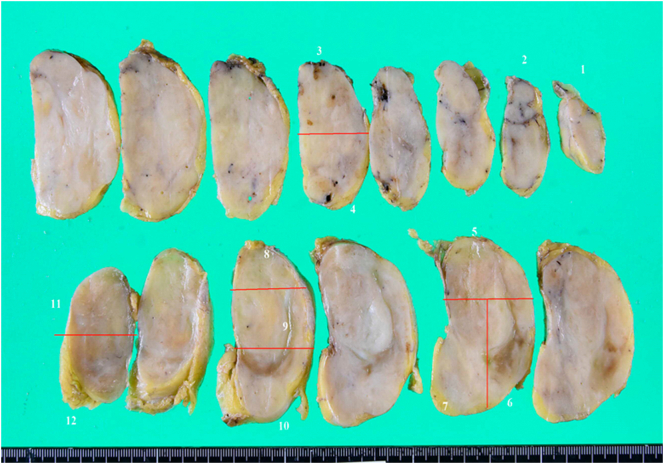
The tumor had a well-defined border with the surroundings and was covered with a fiber coat.

**Fig. 10:**
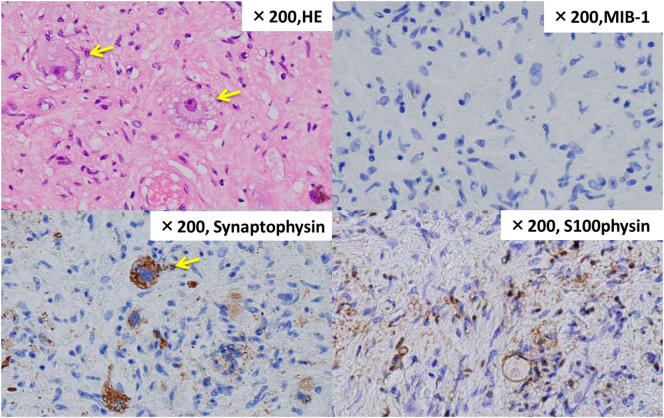
There were spindle-shaped cells with wavy nuclei and scattered ganglion cells on HE staining. Nuclear atypia and mitotic figures were not conspicuous, MIB-1 was less than 1%, no malignant findings were observed.

The size of resected tumor was 110 × 70 mm. A pathological examination of the tumor confirmed the diagnosis of ganglioneuroma with clear surgical margins (Fig. 8, Fig. 9, Fig. 10). Since the patient recovered without complications, she was discharged on the 8th day after surgery. At the time of writing this report, the patient has shown no signs of recurrence and has been undergoing regular follow-up examinations as an outpatient for 1 year since resection.

Written informed consent was obtained from the patient for the publication of the present case report and any accompanying images.

## 3 Discussion

Ganglioneuroma is a sympathetic nervous system tumor derived from neural crests and is a relatively rare disease that accounts for 0.7–1.8% of primary retroperitoneal tumors [2]. Sympathetic nervous system tumors are classified into neuroblastoma, ganglioneuroblastoma, and ganglioneuroma, according to the degree of differentiation. Ganglioneuroma is a highly differentiated benign tumor. The age of onset is <20 years of age in 40% of patients, 20–40 years of age in 40% of patients, and ≥ 40 years of age in 20% of patients [3]. At, 1.13:1, the sex ratio is approximately equal [4]. This tumor can originate from any sympathetic nervous system tissue in the body; however, it is most frequently found in the mediastinum (39%), followed by the retroperitoneum (30%), and adrenal gland (22%); it is also found in the pelvic cavity and neck [5].

Ganglioneuroma is often asymptomatic and is often found by chance during imaging examinations, such as those performed in medical examinations. Pain, including abdominal pain and lower back pain (64%), is the most frequent symptom, followed by gastrointestinal symptoms (16%) (e.g., palpable mass (18%), nausea, vomiting, and anorexia) [6,7]. In this case, gastrointestinal symptoms, such as nausea, vomiting, and anorexia were the patient's chief complaints. Although ganglioneuroma is endocrine-inactive, some ganglioneuromas produce symptoms such as diarrhea, hyperhidrosis, flushing, and hypertension due to the production of catecholamines and vasoactive intestinal peptides (VIP) [8]. On imaging, CT often shows homogenous low density mass, while contrast-enhanced CT shows poor contrast effects. It has been reported that punctate calcification is observed in approximately 20% of cases. MRI shows a uniform low signal on T1WI, which is correlated with the presence of a mucous matrix in the tumor on T2WI; areas with a large amount of mucus matrix a high signal intensity, while those with a large amount of ganglion cells and fiber components show a low signal intensity. In addition, the contrast effect is poor in the early phase of the contrast, and the contrast effect is gradually enhanced in the late phase [[9], [10], [11]]. For the treatment of ganglioneuroma, complete resection by surgery is basically recommended. It has been reported that metastasis to malignant tumors, such as ganglioneuroblastoma, neuroblastoma, and mixed cases are rare but exist, and an accurate diagnosis cannot be made and malignancy cannot be ruled based on the imaging findings alone. In addition, it has been reported that 64.7% of cases diagnosed as adrenal ganglioneuroma after surgery had a different imaging diagnosis before surgery. In this case, a desmoid tumor was suspected based on the imaging findings before the operation; however, the pathological examination after the operation led to the diagnosis of ganglioneuroma.

Regarding the method of surgery for retroperitoneal ganglion cell tumors, there have been recent reports of cases treated by laparoscopic surgery are found. We searched for the key words ‘retroperitoneum, ganglioneuroma, laparoscopic’ and found 13 cases, including our case. In addition, we identified 13 cases in which laparoscopic surgery was used to treat retroperitoneal ganglioneuroma. In general, it is reported that laparoscopic surgery is appropriate for tumors of <7 cm in diameter. The tumor in our case was the largest to be treated by a totally laparoscopic approach (Table 1).

**Table 1:** Clinical characteristics of laparoscopic resection of retroperitoneal mass in 13 patients.

	Author	Year	Sex	Age	Preoperative diagnosis	Size (mm)	Postoperative diagnosis
1	Abe	2006	F	22	Non-functional adrenal tumor		Ganglioneuroma
2	Sasaki	2010	F	69	Retroperitoneal tumor	55 × 38	Schwannoma
3			F	22	Retroperitoneal tumor	40 × 40	Ganglioneuroma
4			F	31	Retroperitoneal tumor	50	Ganglioneuroma
5	Kaneko	2011	F	20	Retroperitoneal tumor	15	Ganglioneuroma
6	Yagisawa	2013	F	29	Retroperitoneal tumor	50 × 100 × 70	Ganglioneuroma
7	Tsutsui	2013	F	38	Ganglioneuroma	35 × 20	Ganglioneuroma
8	Nakano	2013	M	40	Retroperitoneal tumor	52 × 47 × 23	Ganglioneuroma
9	Yoshida	2014	F	32	Pheochromocytoma	63 × 39	Ganglioneuroma
10	Ito	2014	F	35	Non-functional adrenal tumor	90	Ganglioneuroma
11	Nakano	2016	F	30代	Giant retroperitoneal tumor	155	Ganglioneuroma
12	Yamasaki	2017	M	59	Adrenal tumor and ganglioneuroma	30 10	Metastasis of MTC and ganglioneuroma
13	Our case		F	40	Desmoid tumor	70 × 110	Ganglioneuroma

The tumor in our case exceeded 10 cm. It was suggested that adaptation of laparoscopic surgery should be considered based on the observation of organ invasion or vessel invasion and adhesion around the tumor, rather than based on the diameter of the tumor. This case report demonstrates that total laparoscopic surgery is a safe and feasible approach for the treatment of less invasion retroperitoneal ganglioneuroma.

## 4 Conclusion

The present study reports the case of a patient with a retroperitoneal ganglioneuroma of >10 cm in size that was successfully resected by totally laparoscopic approach. This approach is less invasive than conventional laparotomy methods and achieves good cosmetic outcomes. Thus, totally laparoscopic procedures should be considered more often for the treatment of retroperitoneal tumors.

## Abbreviations

CT: computed tomography
MRI: magnetic resonance imaging
T2WI: T2 weighted image
PET-CT: positron emission tomography CT
FDG: fluorodeoxyglucose
SUV: standardized uptake value

## Consent for publication

Written informed consent was obtained from the patient for the publication of this case report and its accompanying images. A copy of the written consent is available for review by the Editor-in-Chief of this journal on request.

## Funding statement

This research did not receive any specific grant from funding agencies in the public, commercial, or not-for-profit sectors.

## Declaration of competing interest

The authors declare that they have no competing interests.
